# The neuroenergetics of stress hormones in the hippocampus and implications for memory

**DOI:** 10.3389/fnins.2015.00164

**Published:** 2015-05-06

**Authors:** Danielle M. Osborne, Jiah Pearson-Leary, Ewan C. McNay

**Affiliations:** ^1^Behavioral Neuroscience, University at AlbanyAlbany, NY, USA; ^2^Department of Anesthesiology and Critical Care Medicine, Children's Hospital of PhiladelphiaPhiladelphia, PA, USA; ^3^Biology, University at AlbanyAlbany, NY, USA

**Keywords:** glucose, hippocampus, glutamate, memory, glucose transporters, epinephrine, glucocorticoids, norepinephrine

## Abstract

Acute stress causes rapid release of norepinephrine (NE) and glucocorticoids (GCs), both of which bind to hippocampal receptors. This release continues, at varying concentrations, for several hours following the stressful event, and has powerful effects on hippocampally-dependent memory that generally promote acquisition and consolidation while impairing retrieval. Several studies have characterized the brain's energy usage both at baseline and during memory processing, but there are few data on energy requirements of memory processes under stressful conditions. Because memory is enhanced by emotional arousal such as during stress, it is likely that molecular memory processes under these conditions differ from those under non-stressful conditions that do not activate the hypothalamic-pituitary-adrenal (HPA) axis. Mobilization of peripheral and central energy stores during stress may increase hippocampal glucose metabolism that enhances salience and detail to facilitate memory enhancement. Several pathways activated by the HPA axis affect neural energy supply and metabolism, and may also prevent detrimental damage associated with chronic stress. We hypothesize that alterations in hippocampal metabolism during stress are key to understanding the effects of stress hormones on hippocampally-dependent memory formation. Second, we suggest that the effects of stress on hippocampal metabolism are bi-directional: within minutes, NE promotes glucose metabolism, while hours into the stress response GCs act to suppress metabolism. These bi-directional effects of NE and GCs on glucose metabolism may occur at least in part through direct modulation of glucose transporter-4. In contrast, chronic stress and prolonged elevation of hippocampal GCs cause chronically suppressed glucose metabolism, excitotoxicity and subsequent memory deficits.

## Introduction

Although chronic stress can impair memory acquisition and consolidation, mild acute stress can enhance these processes. The effect of acute stress on memory facilitation generally follows an inverted-U dose-response curve, similar to that observed with other conditions or treatments that modulate memory (such as administration of glucose; Parsons and Gold, 1992; Baldi and Bucherelli, 2005; Sunram-Lea et al., 2011). Stressful events can produce long-lasting and powerful memories, which in some cases may form the basis for post-traumatic stress disorder (Elzinga and Bremner, 2002; Debiec and LeDoux, 2006; Johnson et al., 2012). Many molecular events initiated by stress, regulated primarily by the hypothalamic-pituitary-adrenal axis (HPA), converge on memory systems in the brain to regulate memory processing (Elzinga and Bremner, 2002). This regulation includes provision and utilization of metabolic fuels, such as glucose and lactate, likely to sustain the increased neuronal energy demands of glutamatergic and catecholamine neurotransmission that occur during learning. The metabolic actions of molecules released by the HPA axis during the stress response are well-characterized peripherally, where they act to mobilize energy stores. However, less is known about their effects on brain energy metabolism. Intriguingly, although both norepinephrine (NE) and glucocorticoids (GCs) levels increase in the brain following stress, NE and GCs have opposing effects on glucose metabolism: NE rapidly enhances glucose metabolism (Fillenz et al., 1999; Gibbs et al., 2008a), while GCs produce a delayed decrease in glucose metabolism (Sapolsky, 1986). We suggest that this timeline of metabolic regulation is critical to memory modulation by stress.

Motivated by research demonstrating that hormones in the stress response have distinct effects on neural energy metabolism, this review focuses on how changes in stress hormones and the timing of their release facilitates the consolidation of stress-related memories. Enhancing hippocampal metabolism (e.g., by direct provision of exogenous glucose) can improve memory formation, while interfering with hippocampal energy metabolism impairs memory. As such, we focus on the hippocampus and how NE and GCs work in concert to affect hippocampal-dependent memory formation. This review will focus on animal work, which primarily utilizes tasks that elicit a fear response (e.g., contextual/cued fear, inhibitory avoidance). Although stress and fear are not interchangeable, inducing stress in animals without a fear component presents several challenges; in particular, inducing stress by food deprivation is potentially confounding for studies of metabolism. Tasks which induce fear are extremely well validated in their ability to activate the HPA axis and function as a stressor, but it is not our goal to suggest that these behaviors are synonymous. Additionally, as the impact of stress hormones varies by brain region and neural system, discussion will include other primary regions involved in memory circuits such as the medial prefrontal cortex and basolateral amygdala. However, it is outside the scope of this review to discuss effects of NE and GCs on these regions in the level of detail that we will use for the hippocampus.

The overall hypothesis being suggested here, therefore, is that many of the effects that stress molecules exert on memory may be mediated through their ability to alter brain metabolism. Specifically, we hypothesize that the timeline for the effects of these hormones in the hippocampus indicates that NE likely enhances hippocampal glucose utilization (Fillenz et al., 1999; Gibbs et al., 2008a), while GCs counteract the effects of NE by decreasing hippocampal glucose uptake (Sapolsky, 1986) and metabolism as a means of preventing excitotoxicity or encoding of competing information. There are several overlapping mechanisms through which NE and GCs affect neural energy metabolism. We focus on regulation of energy mobilization, utilization, and control in the hippocampus, and how these actions may potentiate memory consolidation of stressful stimuli.

## Overview of stress responding and energy metabolism

### Stress-response axis overview

A defining feature of stress is activation of the HPA axis, which prompts secretion of epinephrine from the adrenal medulla (Womble et al., 1980). Epinephrine stimulates central afferents which connect to the locus coeruleus to trigger release of NE in various regions including the hippocampus, basolateral amygdala and medial prefrontal cortex (Wong et al., 2012). NE projections to the hypothalamus, as well as inputs from the central amygdala and bed nucleus of the stria terminalis to the paraventricular nucleus of the hypothalamus, initiate the stimulation and release of corticotropin releasing hormone from the paraventricular nucleus (Petrov et al., 1993). Corticotropin releasing hormone, as well as vasopressin from the hypothalamus, stimulates the release of pro-opiomelanocortin, which is then cleaved to various products including adrenocorticotropic hormone within the anterior pituitary. Adrenocorticotropic hormone is the primary stimulator of GC synthesis. Adrenocorticotropic hormone is released into the blood stream from the pituitary and stimulates steroidogenesis in the adrenal cortex to produce corticosterone in rodents, cortisol in humans (collectively referred to as GCs herein). Once released from the adrenals, within minutes (peaking at 8–10 min), GCs reach the hippocampus via the bloodstream (Barbaccia et al., 2001). Although both of the two GC receptor types, mineralocorticoid (MRs) and glucocorticoid receptors (GRs), are present throughout the brain, the hippocampus has the highest level of receptor co-localization in the brain (Sarrieau et al., 1984; Reul and de Kloet, 1985, 1986; Van Eekelen et al., 1988; Herman et al., 1989; Decavel and Van den Pol, 1990; Funder, 1994; Cullinan, 2000; Reul et al., 2000a,b; Barbaccia et al., 2001). Activation of GRs by GCs is necessary for negative feedback termination of the HPA response in several brain regions, including the cortex, hippocampus, and paraventricular nucleus. In addition to its importance in mediating aspects of learning and memory, the hippocampus is a key region for studying the effects of stress on energy metabolism because it both receives noradrenergic input from the locus coeruleus and is highly sensitive to all the diverse actions of GCs.

### Memory formation under stress

Both human and animal studies have demonstrated a facilitative role of acute stress on formation of powerful and long-lasting memories (Oitzl and de Kloet, 1992; Sandi and Rose, 1994a,b; Roozendaal et al., 1996; Sandi et al., 1997; Oitzl et al., 2001; Lupien et al., 2002a,b; Cahill et al., 2003; Cahill and Alkire, 2003). Stress modulates memory formation as a consequence of bringing “online” the paraventricular nucleus, basolateral amygdala, locus coeruleus, and other networks involved in the stress response (de Kloet et al., 2005). Through modulation of afferents from these networks, NE and GCs create a unique biochemical milieu in the hippocampus (McEwen, 2001) conducive to enhanced hippocampal-dependent learning and memory.

Early in the stress response NE is released into several memory areas of the brain, with effects in the basolateral amygdala being critical for memory consolidation following acute stress (Ferry and McGaugh, 1999; Hatfield and McGaugh, 1999; LaLumiere et al., 2003; Lalumiere and McGaugh, 2005). Lesions of the basolateral amygdala block NE's ability to facilitate cognitive enhancement (Liang and McGaugh, 1983; Liang et al., 1990; Roozendaal and McGaugh, 1996). Blocking adrenergic receptors in the amygdala also blocks memory enhancement by stress (Liang et al., 1986; Quirarte et al., 1997), confirming the essential role of amygdala NE in stress-related memory enhancement; similarly, β-adrenergic receptor antagonist infusion into the basolateral amygdala attenuates the memory enhancing effects of GR agonism administered immediately after inhibitory avoidance training (Roozendaal et al., 1999). While the memory enhancing effects of NE in the basolateral amygdala are well-established, the effects of NE in the hippocampus are less clear. The hippocampus is vital for formation and storage of stress-related memories, but requires additional input from the amygdala in order to form a highly salient, vivid memory (McGaugh, 2004; Roozendaal et al., 2004). Stressful stimuli produce synchronized theta activity in both the amygdala and hippocampus, suggesting that the two structures are directly engaged while processing stressful stimuli (Pare et al., 2002; Moita et al., 2003; Seidenbecher et al., 2003; Pape et al., 2005; Narayanan et al., 2007). Furthermore, mRNA for *Arc* in the dorsal hippocampus is increased following β2 adrenergic agonism administered into the basolateral amygdala at levels commensurate with those found following spatial training (Guzowski et al., 2001; Vazdarjanova et al., 2006; Miyashita et al., 2009); *Arc* is an immediate-early gene product specifically linked to LTP and memory formation (Guzowski et al., 1999, 2006; Vazdarjanova et al., 2002; Ramirez-Amaya et al., 2005; Holloway and McIntyre, 2011). NE also has effects that are specific to the hippocampus: projections from the locus coeruleus release NE directly in the hippocampus where it acts to enhance hippocampal-dependent learning. Infusion of NE into the dorsal hippocampus increases contextual fear learning (Yang and Liang, 2014), and directly regulates the calcium-responsive element binding protein CREB, which is a central component of hippocampal memory formation (Kabitzke et al., 2011; Morris and Gold, 2012). NE effects in the hippocampus are believed to be largely due to NE binding to β-adrenergic receptors (βARs): intraperitoneal administration of the βAR antagonist propranolol prior to or following training inhibits spatial cognition and contextual fear recall (Stuchlik et al., 2009; Kabitzke et al., 2011). NE has effects in both the amygdala and hippocampus to enhance stress-related learning, and these effects may be differentially regulated by specific NE receptor subtypes (Mueller et al., 1981; Quirarte et al., 1997; Pearson and Frenguelli, 2004; Roozendaal et al., 2006; Gibbs et al., 2008a; Hutchinson et al., 2008; McReynolds et al., 2010). One element of the interaction between amygdala and hippocampus in formation of stress-related memories is that amygdala stimulation facilitates hippocampal LTP: this facilitation is both GC- and NE-dependent (Quirarte et al., 1997; Ferry et al., 1999; Roozendaal et al., 2006; McReynolds et al., 2010; McGaugh, 2013).

In contrast to NE, the actions of GCs are more diverse and do not always facilitate learning and memory. GCs can affect a wide range of hippocampal processes depending on the timing and dose of exposure. Timing of exposure is crucial regarding whether GCs will enhance or inhibit memory formation. Animal and human studies have shown that exposure to GCs during learning facilitates memory, while exposure after learning or around retrieval has the opposite effect (Oitzl and de Kloet, 1992; Kirschbaum et al., 1996; Sandi and Rose, 1997; Oitzl et al., 2001; Roozendaal, 2002; Joels, 2006). Additionally, if GC stimulation/administration occurs hours prior to NE secretion, it will inhibit the memory facilitating effects of NE (Borrell et al., 1984; Joels and de Kloet, 1989; Roozendaal, 2003; Richter-Levin, 2004). This time-dependency is true *in vitro* also, where GCs can facilitate LTP only if administered around the time that LTP is induced (Joels, 2006). GC dosage will further influence the effects on cognitive functions. Different doses of GCs are associated with different patterns of gene expression in the hippocampus (Polman et al., 2013), with the CA1 region in particular having a U-shaped dose response to GCs (Joels, 2006). Like many cognitive modulators (e.g., glucose or insulin), physiological-level and temporary moderate increases in GCs are key to eliciting enhancements in hippocampal-dependent learning and memory. In brief, complete removal of GCs by adrenalectomy hampers cell function and consolidation of stressful events, moderate doses of GCs will typically enhance learning (timing-dependent), while high doses will impair consolidation and recall (Joels, 2006; Spanswick et al., 2011). Chronic exposure to GCs can be particularly detrimental. Administration of high and/or chronic levels of GCs produces cognitive deficits and even hippocampal atrophy (Sapolsky et al., 1985; Sapolsky, 1996; Joels et al., 2004; Yun et al., 2010). Chronic GCs will also induce metabolic dysfunction and insulin resistance (eventually leading to type II diabetes mellitus) which can result in further damage to hippocampal structure, physiology, and function (Willi et al., 2002; Stranahan et al., 2008; Karatsoreos et al., 2010; Ye et al., 2011; Reagan, 2012).

### Stress effects on glutamate

**Figure 1:**
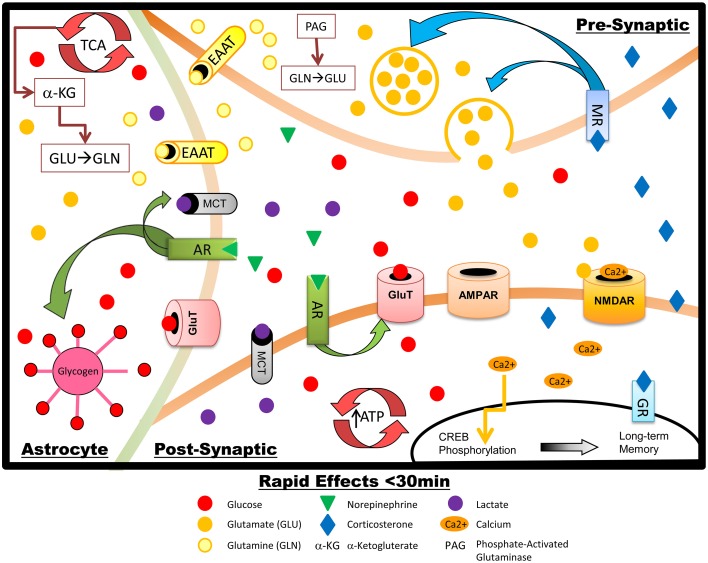
**Schematic summary of the rapid effects of stress hormones on metabolism and glutamate transmission in hippocampal neurons and astrocytes.** NE increases glycogen breakdown in astrocytes, with subsequent lactate release. NE also upregulates GluT translocation in the post-synaptic neuron, increasing glucose transport, energy production, and firing during memory processing: increased glucose metabolic demand is likely driven primarily by restoration of the neuronal membrane potential after glutamate-induced depolarization. AR activation also promotes phosphorylation of CREB which increases LTP and memory formation. Rapid effects of GCs include upregulation of presynaptic glutamate synthesis and release.

**Figure 2:**
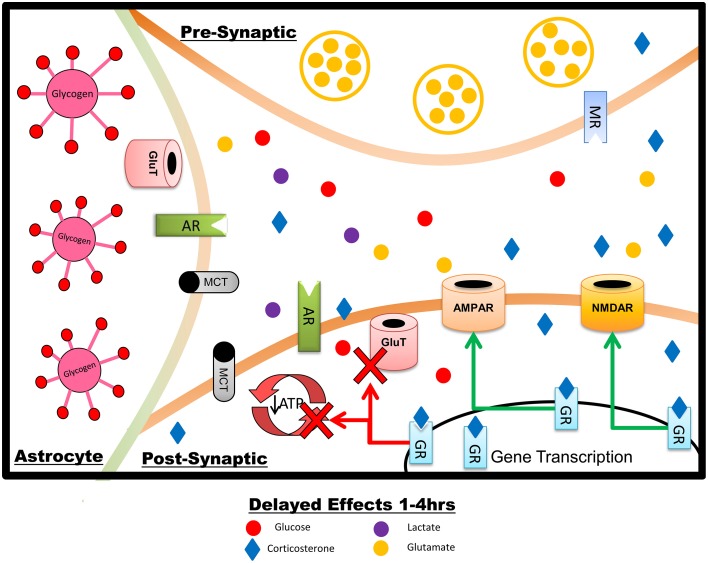
**Summary of the delayed effects of stress on hippocampal neurons and astrocytes, reflecting primarily genomically-mediated effects of GCs.** Over the course of minutes to hours after a stressor exposure, GCs increase AMPAR and NMDAR insertion into the post-synaptic membrane but reduce plasma membrane GluTs. Glucose transport and metabolism are downregulated in astrocytes and neurons, production and metabolism of lactate is lowered, and expression of prometabolic genes is reduced. The overall effect, under normal physiological circumstances, is to downregulate both neuronal firing and metabolism, acting to reverse the short-term impact of stress and promote optimal memory for stress-related events with minimal or no cellular damage. The increase in glutamate receptors may potentiate the response to subsequent or prolonged stress, potentially including excitotoxic damage.

One route by which stress may facilitate learning is by altering glutamatergic neurotransmission, which can occur following HPA activation of GCs (Holmes and Wellman, 2009). Following peripheral corticosterone administration, glutamate release significantly increases in the hippocampus while adrenalectomy can decrease basal levels of glutamate in the hippocampus, an effect ameliorated by administration of corticosterone (Venero and Borrell, 1999). Similar increases in glutamate release in the hippocampus follow either 7 days of restraint stress (Zhu et al., 2008) or acute ether stress (Abraham et al., 1998) and adrenalectomy again abolished the elevation in excitatory amino acids. Several mechanisms have been suggested to transduce the effect of GCs on hippocampal glutamate release. Presynaptically, these include an increase in SNARE related proteins (Musazzi et al., 2010), increased vesicular glutamate transporter-2 in the hippocampus (Lussier et al., 2013) and non-genomic effects through a presynaptic membrane-bound MR that increases the probability of glutamate release by activating ERK1/2 (de Kloet et al., 2008), shown in Figure 1. GCs also regulate postsynaptic glutamate receptors, as shown in Figure 2. Corticosterone or dexamethasone administration increases both AMPAR and NDMAR synaptic currents and translocation actions in the medial prefrontal cortex (Yuen et al., 2011), while the impact of GCs in the hippocampus appears to be primarily on AMPAR (Karst and Joels, 2005; Yuen et al., 2009). *In vitro*, corticosterone increases trafficking of GluR2-containing AMPARs to the synaptic membrane (Groc et al., 2008; Martin et al., 2009), while stressful watermaze training increases *in vivo* synaptic expression of GluR2-containing AMPA receptors (Conboy and Sandi, 2010). The rapidity of these effects suggests a non-genomic mechanism. However, patch-clamp recordings from PFC slices indicate that GCs also have prolonged effects on glutamatergic signaling, suggesting that both genomic and non-genomic mechanisms may promote glutamate release and upregulated activity of postsynaptic glutamate receptors (Musazzi et al., 2010). The multiple mechanisms by which GCs increase glutamatergic signaling are likely to support increased LTP, and hence memory formation. The metabolic cost of these actions is substantial, however.

Neuronal activity that causes glutamate release and post-synaptic activation of glutamate receptors requires large quantities of energy (Harris et al., 2012). Around 55% of total ATP consumption for action potentials in the brain is used at the synapse, largely for glutamate transmission (Attwell and Laughlin, 2001): roughly 40% of synaptic energy consumption fuels glutamate (NMDAR and non-NMDA) receptor activity, including restoration of membrane potentials (Attwell and Laughlin, 2001; Harris et al., 2012). In addition to energy costs associated with glutamate activity at its receptors, glutamate synthesis may also account for up to 20% of glucose utilization (Hertz, 2006). Astrocytes play an active role in glutamine-glutamate recycling (Figure 1), glutamate synthesis, and oxidative degradation of glutamate: all of these processes further add to the glycolytic cost of glutamate activity (Hertz, 2006; Gibbs et al., 2008b). A further use of fuel to support glutamatergic activity comes from glycogenolysis in astrocytes, which is required for glutamate production (Gibbs et al., 2008b; Hertz et al., 2014a, 2015). Because one of the key effects of GCs in the brain is to increase metabolically-demanding glutamatergic activity, we will detail how GCs working in concert with NE may affect brain glucose metabolism in order to meet the high energetic demands of stress-associated learning. In some cases the modulatory effects that these molecules exert on brain energy metabolism differ from effects in the periphery (Pagano et al., 1983; McMahon et al., 1988; Garvey et al., 1989; Oda et al., 1995; Battelino et al., 1996; Sakoda et al., 2000). Adequate supply of glucose for close regulation of glutamatergic transmission is especially important, compared to that of other neurotransmitters, because unregulated glutamate release and activity will not only impair the formation of specific memories but can also lead to cellular damage including apoptosis (Mohseni, 2014). Perturbation of the Krebs cycle, changes to mitochondrial membrane potential, and accumulation of reactive oxygen species (Hernandez-Fonseca et al., 2008; Sutherland et al., 2008; Stelmashook et al., 2010) all occur under conditions where glucose supply is impaired (e.g., stroke, hypoglycemia, Mattson, 1997; Martin et al., 1998; Mohseni, 2014); under such conditions removal of glutamate from the synapse is impaired (Camacho and Massieu, 2006) which leads to excitotoxicity.

## Metabolism and memory: emphasis on interaction with stress

Optimal hippocampal glucose supply and utilization is critical for memory formation (Gold, 1986; Lee et al., 1988; Krebs and Parent, 2005; Suzuki et al., 2011). Administration of glucose, either peripherally or directly to the hippocampus, can improve memory in a dose-dependent manner (Messier, 2004). Decreased brain glucose supply during hypoglycemia impairs memory (Korol and Gold, 1998; Messier, 2004), and conditions that impair brain glucose metabolism (such as type 2 diabetes, or Alzheimer's disease) also cause cognitive impairment (Moroz et al., 2008; de la Monte, 2012). Memory formation is limited by hippocampal glucose supply: cognitively demanding tasks rapidly deplete hippocampal extracellular glucose levels by an amount correlated with task difficulty, while doses of glucose that prevent such depletion also enhance memory (McNay et al., 2000). These data suggest that glucose is a limited commodity in the hippocampus whose availability directly regulates the ability to form memories. Tangentially, similar glucose supply-limited performance is also seen on tests of e.g., self-control (Gailliot and Baumeister, 2007; Gailliot et al., 2007; Job et al., 2013) mediated largely by the medial prefrontal cortex, supporting glucose supply as a key regulator of cognitive performance across cognitive domains and brain regions; direct measurement of glucose depletion in the amygdala during a fear avoidance task also supports the generality of glucose metabolism as a key regulator of memory formation across brain regions (Sandusky et al., 2013).

How exactly increased glucose metabolism facilitates superior hippocampal memory formation is still unclear, but several mechanisms have been discussed including e.g., provision of substrate for neurotransmitter synthesis or metabolic support for glial regulation of glutamate within synapses (McNay and Gold, 2002). It is likely that glucose permits and supports multiple memory processes; even in the absence of stress, increased glucose metabolism is associated with increased neural activity and forms the basis for modern techniques aimed at measuring neural activity, such as PET and fMRI in humans and 2-deoxyglucose (2DG) in animals. The mechanisms coupling neuronal activity to glucose metabolism have been the subject of debate since at least the seminal work of Roy and Sherrington (Roy and Sherrington, 1890), and more recently it has been suggested that under some circumstances neurons may uptake fuel in the form of lactate coming from glucose metabolism in astrocytes, rather than always metabolizing glucose directly themselves (Pellerin et al., 1998, 2007), consistent with the suggestion that memory facilitation by provision of additional glucose may be transduced, at least in part, by glial mechanisms (McNay and Gold, 2002). For the purposes of this review, the question of glial vs. neuronal glucose use is largely irrelevant: undoubtedly both cell types contribute to processes underlying memory formation.

A key component of the body's response to stress is to mobilize energy stores and direct energy toward processes necessary for fight or flight responses (Livingston and Lockwood, 1975; De Bruin et al., 1990; Weissman, 1990). Preservation of glucose supply to the brain is a priority: neuroglycopenia triggers a stress response even if systemic glucose levels are normal (Borg et al., 1995, 1997, 2003). Whether triggered by hypoglycemia or another stressor, activation of the HPA axis and subsequent release of GCs and NE causes release of glucose from hepatic stores, primarily to ensure that brain glucose supply is maintained (McMahon et al., 1988; Hoffman, 2007). Thus, systemic GCs can affect brain glucose supply by increasing systemic concentration of glucose. During acute stress, glucose utilization rapidly increases in the hippocampus (Schasfoort et al., 1988) even above the normally high metabolic rate: the brain is hence a major consumer of glucose during stress, and the fact that the brain has only minimal energy reserves makes it critically dependent on supply from the periphery. Acute stress produces a large rise in peripheral glucose, which will enhance memory formation in the same way as exogenous glucose administration (Munck, 1971; Zyskowski and Munck, 1979); increases in blood glucose are rapidly reflected in increased brain glucose supply (Silver and Erecinska, 1994; McNay et al., 2001; McNay and Sherwin, 2004). The surge in blood glucose levels during stress could, therefore, enhance hippocampally-dependent memory; as noted above, the inverted-U dose-response curve for modulation of memory by stress resembles that for modulation by glucose.

The fact that stress increases glutamatergic signaling in the hippocampus likely facilitates enhancement of memory processing, but also increases both the need for glucose to support neuronal transmission and the potential risk if glucose supply is inadequate. When supply of glucose is insufficient to support regulation of glutamatergic signaling and subsequent calcium influx through NMDAR, excitotoxicity ensues (Martin et al., 1994). Consistent with this, chronic stress can induce excitotoxicity via prolonged upregulation of GCs (Popoli et al., 2012). Glucose administration to cells exposed to GCs can prevent the excitotoxic effects of excitatory amino acids (Sapolsky, 1986). Hence, mobilization of glucose stores during stress likely acts in concert with elevated brain glutamatergic neurotransmission to enhance memory processes by allowing enhanced energy provision to the hippocampus, permitting increased glutamatergic transmission without risk of cell loss.

In addition to increasing glucose, stress also produces a large rise in hippocampal lactate, which can occur within minutes of the induction of stress (De Bruin et al., 1990; Krugers et al., 1992; Fellows et al., 1993). The rapidity of this rise in hippocampal lactate during the stress response suggests that elevated hippocampal lactate is supplied by release of lactate from active astrocytes or neurons following glucose metabolism (Hertz et al., 2007); this is supported by the finding at times of cognitive demand, the initial rise in hippocampal lactate is in proportion to increased local glucose metabolism (McNay and Sherwin, 2004; Newman et al., 2011). Lactate may aid in supporting sustained activation of hippocampal neurons involved in memory (Magistretti and Pellerin, 1999; Allaman et al., 2011; Newman et al., 2011; Suzuki et al., 2011). The relative contribution of glucose and lactate to neuronal metabolism at times of high neuronal activation is a topic of active research. A leading hypothesis is that there may be a contribution of astrocyte-derived lactate to neuronal metabolism (an “astrocyte-neuron lactate shuttle”) under at least some conditions (Pellerin et al., 1998; Bouzier-Sore et al., 2003; Ros et al., 2005; Wyss et al., 2011). The impact of stress on such a shuttle, if any, is not yet determined, but results to date are consistent with a possible role for a lactate shuttle in contributing to metabolic support for stress-induced memory processing. Astrocytes are capable of supplying both glucose and lactate to neurons, and the shuttling rate for lactate between astrocytes is significantly greater than the rate at which astrocytes can provide lactate to neurons (Gandhi et al., 2009); an alternative hypothesis to that of a direct lactate metabolic shuttle is that the impact of lactate production on neuronal activity may be at least in part via mechanisms other than direct metabolic utilization, with glucose remaining the primary fuel utilized by neurons (Gibbs et al., 2007; Dienel, 2013; Hertz et al., 2014b). By increasing the supply of both glucose and lactate to the hippocampus, stress hormones may hence synergistically facilitate the encoding of memories during stress via multiple channels of metabolic support. Below we review evidence for differential modulation of energy metabolism in the brain by NE and GCs.

### Rapid effects of norepinephrine

As discussed above, the rapid rise in systemic epinephrine caused by stress immediately increases circulating glucose via release of hepatic glycogen stores (Gold, 2014). A second major effect of this epinephrine increase, occurring on a similar timescale, is stimulation of the vagal nerve: in addition to peripheral effects such as an increase in heart rate (and hence blood supply to the brain), stimulation of the vagal nerve directly triggers release of NE in the amygdala via synapses in the nucleus of the solitary tract (Miyashita and Williams, 2003, 2004, 2006; Hassert et al., 2004) and this stimulation alone can enhance memory formation at times of stress, via NE transmission in the amygdala (Miyashita and Williams, 2002, 2003; Pena et al., 2013, 2014). The combination of these two effects means that epinephrine both stimulates hippocampal stress-modulated memory processes *and* increases glucose supply to meet the demands of these processes.

Much of the work on neuromodulatory effects of NE has focused on NE as a regulator of other neurotransmitter systems, such as modulation of glutamatergic neurotransmission (Ferry et al., 1997). In addition, however, a stress-induced rise in central NE could act in synergy with peripheral epinephrine to further increase hippocampal glucose metabolism and hence support the metabolic demands of increased (glutamatergic) activity in the hippocampus. Concurrent with the rise in hippocampal extracellular glucose during stress, NE levels in the hippocampus also increase (Schasfoort et al., 1988; De Bruin et al., 1990; Tajima et al., 1996). Briefly, there are at least three ways in which this rise in NE increases glucose metabolism in the CNS (shown in Figure 1). First, NE increases astrocytic glycogenolysis leading to increased astrocytic metabolism and production of lactate. Secondly, NE can trigger neural glycolysis. Thirdly, NE can increase neuronal and astrocytic glucose transporters at the cell surface to increase glucose throughput. Importantly, these effects occur within a matter of minutes of stress, systemic epinephrine increase, and subsequent NE release. These mechanisms are discussed in detail in the following paragraphs.

Work largely from Marie Gibbs' group has produced detailed understanding of how NE plays a central role in modulation of hippocampal memory processes via regulation of local neural metabolism (Hutchinson et al., 2008). Using a day-old chick model, the group has shown that both α-adrenergic and βARs receptors are involved in memory processing, with distinct effects on metabolism (Hourani et al., 1990). For instance, memory enhancement by stimulation of β2-adrenergic receptors (β2-AR) increases astrocytic glycogenolysis, and inhibition of glycogenolysis prevents the memory enhancing effects of β2-AR agonism (Hsu and Hsu, 1990). Astrocytic glycogenolysis produces lactate, which (as discussed above) can be shuttled to active neurons and potentially sustain activity during increased metabolic demand, such as observed during acute stress and/or cognitively demanding tasks (Pellerin et al., 2007; Suzuki et al., 2011).

AR activation can stimulate brain glycolysis as well as glycogenolysis. β3-AR agonism stimulates glycolysis in the avian cortex, and administration of the glucose analog 2-deoxyglucose (which competes with glucose for uptake and inhibits glycolysis) prevents the memory enhancing effects of β3-AR agonism (Gibbs et al., 2008a). NE can stimulate glycogenolysis in primary culture of mouse astrocytes, and the effect can be blocked by β-AR antagonism and/or α2-AR antagonism, but not α1-AR antagonism (Subbarao and Hertz, 1990). Hence, both glycogenolysis and glycolysis are involved in and required for noradrenergic memory enhancement. Consistent with this, systemic administration of beta-adrenergic antagonists reduces glycolysis in the human brain (Gam et al., 2009) as well as reducing blood pressure (Arauco and Alagon, 1979; Woods, 1979), and such administration attenuates stress-related memory enhancement (Sternberg et al., 1983; Introini-Collison et al., 1989; Grillon et al., 2004; Lim et al., 2007; Oei et al., 2010; Schneider et al., 2011; Sun et al., 2011; Taherian et al., 2014).

In addition to direct stimulation of glycolysis and glycogenolysis, NE increases hippocampal glucose transport, likely into both astrocytes and neurons. Activation of AR upregulates the intrinsic activity (i.e., transport throughput per transporter) of cell-membrane glucose transporters and also increases translocation of glucose transporters from intracellular storage pools to the plasma membrane (Omatsu-Kanbe and Kitasato, 1992; Shimizu et al., 1998). Time-course analysis suggests that astrocytic/endothelial glucose transporter-1 (GluT1) is necessary for memory consolidation (beginning approximately 30 min post-training), while memory acquisition (occurring during training and immediately afterward) requires a neuronal GluT, such as GluT3 or GluT4 (Gibbs et al., 2008a). GluT3 is a ubiquitously expressed neuronal glucose transporter, but GluT4 has sparser distribution and is particularly elevated in the hippocampus (Vannucci et al., 1998; El Messari et al., 2002); moreover, GluT4 is dynamically and rapidly translocated between the plasma membrane and intracellular storage vesicles in response to both cellular activity and metabolic state (Kristiansen et al., 1996; Hertel et al., 2004; Alkhateeb et al., 2007; Bogan and Kandor, 2010; Ojuka et al., 2012; Pearson-Leary, 2013) whereas GluT3 in neurons is traditionally considered to be constitutively located at the plasma membrane only (although one study suggests a degree of translocation, Uemura and Greenlee, 2006). A role for GluT4 in mediating upregulation of hippocampal glucose metabolism by NE would be highly consistent with findings in peripheral tissues showing that β2-AR agonism can rapidly activate GluT4 translocation (Dehvari et al., 2012), an effect that is regulated by acute metabolic state (Zanquetta et al., 2006). Intriguingly, GluT4 in the hippocampus is considered by many researchers to facilitate glucose utilization specifically during energetically demanding effects such as cognitive demand (McEwen and Reagan, 2004; Bakirtzi et al., 2009), and thus represents a strong candidate as a modulatory glucose transporter to support the energetic demands of memory formation during acute stress. Taken together with the facilitative effects of NE on astrocytic glucose metabolism and glycogenolysis, it is possible that early in the acute stress response NE both activates an astrocyte-neuron lactate shuttle and increases GluT4 activity to prevent insufficient fuel supply for optimal formation of stress-related memories.

In conclusion, NE modulates memory and metabolism concomitantly (Gibbs et al., 2008a) within the limbic system via effects at AR. Moreover, the effect of AR activation on brain metabolism is rapid and primarily occurs during the acquisition and early consolidation phases of memory. The evidence suggests that one effect of NE release in the hippocampus, during stress, is to support the high metabolic demands of stress-enhanced cognitive processing.

### Rapid effects of glucocorticoids

Although this review emphasizes the delayed effects of GCs on hippocampal-dependent memory, it is worth reiterating the rapid effects of GCs on glutamate signaling in the context of timecourse.

Shortly after their release, and in contrast to the delayed effects discussed below, GCs act in concert with NE to further increase hippocampal activity and specifically upregulate glutamate release (shown in Figure 1; Abraham et al., 1998; de Kloet et al., 2008; Lussier et al., 2013). These actions are primarily mediated by presynaptic MRs rather than GRs (de Kloet et al., 2008). GCs have profound and consistent effects to increase glutamate release in the hippocampus, with associated metabolic demand. Importantly, however, administration of GCs to the hippocampus immediately after learning both enhances memory and increases basolateral amygdala levels of NE (McReynolds et al., 2010), suggesting that via a positive feedback relationship with NE, rapid actions of GCs contribute to increasing glucose availability. This feedback loop likely works to ensure adequate metabolic support for the increased hippocampal activity; increased glucose supply is needed both to meet the high energy demands of restoring membrane potential in the aftermath of glutamate-induced depolarization and firing (Attwell and Gibb, 2005) and also as a substrate for increased glutamate synthesis (Attwell and Laughlin, 2001; Harris et al., 2012). These temporally coordinated, synergistic actions create a short window of increased hippocampal activity that likely increases the salience of stress-related memory formation but avoids prolonged elevation in glucose demand.

### Delayed effects of glucocorticoids

GCs are traditionally considered to regulate neural activity primarily via genomic effects, slower than the rapid effects of NE and GCs discussed above. These include modulation of both brain glucose transport and glutamate receptor localization. Modulation of glutamatergic function by GCs includes both regulation of release (discussed above) and both genomic and non-genomic control (Figure 2) over postsynaptic AMPAR and NMDAR activity (Musazzi et al., 2010). GCs can increase AMPAR trafficking *in vitro* (Groc et al., 2008; Martin et al., 2009); *in vivo*, GRs mediate an increase in both AMPA and NMDA receptor current and translocation in the medial prefrontal cortex (Yuen et al., 2011) and similar effects are seen on AMPAR translocation in the hippocampus following either GC administration (Karst and Joels, 2005; Yuen et al., 2009) or stressful water maze training (Conboy and Sandi, 2010).

In both the periphery and the brain, direct regulation of glucose metabolism by GCs is well-documented. In peripheral tissues, GCs stimulate gluconeogenesis and lipolysis, but inhibit glucose uptake (Munck, 1971; Zyskowski and Munck, 1979). Elimination of corticosterone by adrenalectomy increases brain glucose utilization in regions associated with regulation of the stress response, such as the hippocampus, paraventricular nucleus, and locus coeruleus; this effect is reversed by administration of the GR-specific corticosterone analog dexamethasone (Kadekaro et al., 1988) so that the impact of GCs on brain glucose metabolism appears to resemble that on other tissues, with an increase in GCs causing reduced glucose uptake and metabolism.

Although it has not been confirmed whether the effects of GCs on neural metabolism involve gene regulation, the timeline of these effects (generally on the order of minutes to hours, Barbaccia et al., 2001) suggest that this is the case (Abraham et al., 1998; de Kloet et al., 2008; Lussier et al., 2013). Indeed, inhibition of glucose transport by GCs is dependent on protein synthesis (Livingston and Lockwood, 1975). Studies using the glucose analog 2-deoxyglucose have shown that *in vivo*, GCs inhibit glucose metabolism in the hippocampus 4 h following administration. *In vitro*, GC activation of GRs impairs glucose transport into both neurons and glia (Horner et al., 1990; Virgin et al., 1991). Inhibition of MRs does not block the action of GCs on neuronal glucose metabolism (Reagan, 2002). These data suggest that GC-induced inhibition of brain glucose metabolism appears to occur through inhibition of glucose transport, likely at a transcriptional level and via binding to GRs.

GCs can directly inhibit neuronal glucose transport (Piroli et al., 2007), but it is unclear which glucose transporter mediates these effects. GCs have been shown to downregulate GluT3 in the hypothalamus (Cherian and Briski, 2010), but there appears to be little effect of stress on hippocampal GluT3 (Reagan et al., 1999). In peripheral tissues, GCs can impair translocation as well as intrinsic activity of GluT4 (Weinstein et al., 1995; Andrews and Walker, 1999; Sakoda et al., 2000; Jeyaraj et al., 2007), and corticosterone administration downregulates GluT4 expression in the hippocampus (Piroli et al., 2007). It is thus likely that the effect of GCs on glucose transport in hippocampal neurons is mediated through GluT4 rather than GluT3. GCs decrease astrocytic glucose transport (presumably through GluT1) on a time-course of 4 h, similar to the time-course of GC-mediated glucose transport inhibition in neurons (Virgin et al., 1991). Taken together, these data suggest that 4 h of GC exposure is sufficient to inhibit both neuronal and astrocytic glucose transport. The effect of GCs on other metabolic fuel transporters, such as the monocarboxylate transporters responsible for lactate shuttling, are unclear. Overall, though, following a stressful stimulus the impact of GCs on hippocampal glucose metabolism appears to generally oppose the upregulation of metabolism initiated by NE (Figure 2).

Although slightly tangential, it is interesting to note that review of the literature identifies neuronal glucose uptake through GluT4 as a likely transducer of hippocampal memory enhancement by stress-induced metabolic upregulation. A strong prediction from this would be that direct upregulation of hippocampal GluT4 translocation from intracellular vesicles to the neuronal cell surface would correlate with enhanced memory formation. Recent findings from our lab support this hypothesis: intrahippocampal insulin moves GluT4 to the neuronal membrane and enhances both hippocampal memory processes and local glucose metabolism (McNay et al., 2010), while blockade of hippocampal insulin or other treatments that impair GluT4 translocation cause marked memory impairment (McNay et al., 2010; Pearson-Leary and McNay, 2012; Pearson-Leary, 2013); these findings hence offer support for the GluT4-related conclusions of the present review.

GCs have additional effects on metabolism. GR activation decreases neuronal gene expression of genes involved in ATP synthesis, such as F1-ATPase alpha subunit and ATP synthase coupling factor 6 (Morsink et al., 2006). This may account for the accelerated loss of ATP, following metabolic insult, caused by GCs (Lawrence and Sapolsky, 1994). GCs also decrease neuronal gene expression of lactate dehydrogenase B, the predominant neuronal isoform (Bittar et al., 1996; Morsink et al., 2006), which converts lactate into pyruvate, suggesting that GCs can decrease hippocampal neuronal use of lactate as a fuel. Because stress and aversive memory training increases hippocampal extracellular lactate (Schasfoort et al., 1988; Elekes et al., 1996a,b; Korf, 1996; Suzuki et al., 2011), and NE increases lactate production, this is a further case of GCs acting to oppose and reverse the impact of NE on hippocampal metabolism. Beyond the effects of a single stressor, chronic elevation of hippocampal GCs causes vulnerability to excitotoxicity, likely at least in part via down-regulation of glucose metabolism: GCs can directly induce excitotoxicity, and inhibition of glucose transport accelerates GC-induced neurotoxicity (Sapolsky, 1986; Lawrence and Sapolsky, 1994; Roy and Sapolsky, 2003). Conversely, administration of metabolic substrates including glucose reduces hippocampal damage induced by co-administration of GCs and the excitotoxin kainic acid (Sapolsky, 1986). Although beyond the scope of this review, it is likely that the long-term negative cognitive effects of chronic and/or extreme stress are due in large part to a combination of cerebral hypometabolism and cell loss, both mediated by GCs; a contributing factor may be a state of persistently elevated GCs leading to impaired hippocampal glucose metabolism without the compensating effects of NE. Under less pathological circumstances, GC-mediated down-regulation of metabolism when neuronal firing is reduced will be protective, acting to reduce the risk of oxidative damage from excessive metabolism (Vincent et al., 2004; Duarte et al., 2006; Maiese et al., 2007).

In summary, GCs are potent inhibitors of hippocampal metabolism via several mechanisms. Specifically, (i) GCs markedly inhibit glucose transport through GluTs, with regulation of GluT4 in particular a likely transducer of GCs in the hippocampus; (ii) GCs downregulate gene expression of enzymes necessary for glucose metabolism, and (iii) unlike NE, the effects of GCs on brain glucose metabolism are not immediate but rather have a delay of up to 4 h. Overall, GCs have opposing effects to NE: while NE rapidly increases glucose metabolism (i.e., within 10 min of exposure), GCs primarily decrease glucose metabolism on a slower time course.

## Integrated model: concluding remarks

**Figure 3:**
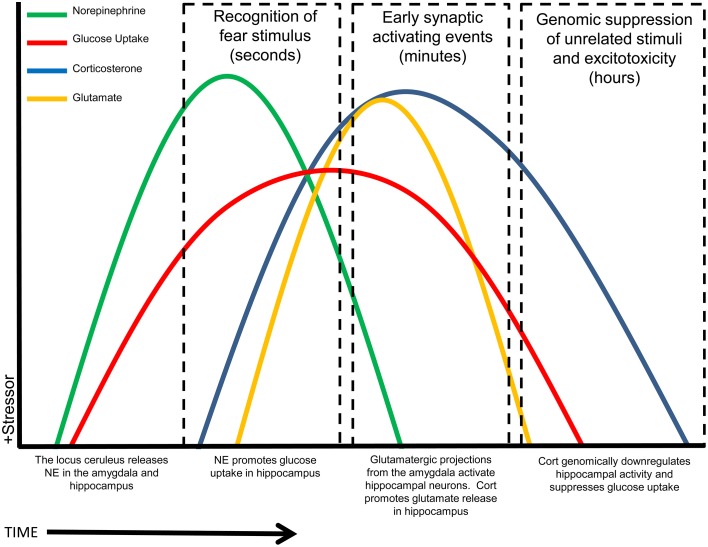
**Summary schematic timeline of stress-related events in the hippocampus.** Rapidly following the recognition of a stressor (time = zero), NE is released and increases glucose release and uptake. Shortly afterward, GCs initially increase glutamate release, but then act to reduce both glucose metabolism and neuronal transmission, restoring a baseline state. X-axis timeline not drawn to scale. NOTE: Some variables may go slightly below baseline at some time points, rather than returning exactly to baseline, including at times after the initial timecourse illustrated here.

The model we suggest, based on the data reviewed here, is that NE transiently enhances hippocampal glucose metabolism, after a stressor, to support the demands of increased stress-related memory processing and specifically glutamatergic signaling in the hippocampus; in contrast, the same stressor triggers release of GCs that act as an “off-switch” and inhibit hippocampal glucose utilization after initial memory formation; this time-course is illustrated schematically in Figure 3. The reversal of upregulated stress-induced glucose metabolism by GCs will have at least two beneficial consequences: it will both reduce the risk of excitotoxicity from increased glutamate release and also, importantly, likely “fine-tune” memory formation by restricting upregulation of memory processes to the immediate aftermath of a stressor, hence increasing the saliency of stress-related events and leading to enhanced memory specifically for the stressful event. We have described a timeline for how NE and GCs may work synergistically to provide necessary fuel for (energetically costly) hippocampally-mediated learning, while minimizing cost and risk to the hippocampus, a structure that is especially vulnerable to excitotoxic damage (Conrad, 2008). Timing and synchronization of activity in the basolateral amygdala and hippocampus with NE and GC release is crucial: we suggest that this modulation of hippocampal glucose provision and metabolism is a critical component of memory modulation by both stress hormones.

### Conflict of interest statement

The authors declare that the research was conducted in the absence of any commercial or financial relationships that could be construed as a potential conflict of interest.
